# Evaluating the Chemical Resistance and Performance of Thermochromic Polymers for Food Packaging

**DOI:** 10.3390/ma18092085

**Published:** 2025-05-01

**Authors:** Colette Breheny, Declan Mary Colbert, Gilberto Bezerra, Joseph Geever, Luke M. Geever

**Affiliations:** Polymer, Recycling, Industrial, Sustainability and Manufacturing (PRISM) Research Institute, Technological University of the Shannon, University Road, N37 HD68 Athlone, Ireland; declan.colbert@tus.ie (D.M.C.); gilberto.bezerra@tus.ie (G.B.); joseph.geever@tus.ie (J.G.)

**Keywords:** thermochromic pigments, food packaging, chemical resistance, thermal cycling, mechanical durability

## Abstract

The use of thermochromic pigments in food packaging offers several advantages, including improved food safety, waste reduction, and temperature change monitoring. However, little is known about how chemically resilient these materials are, especially regarding optical stability, thermochromic activation, and mechanical integrity following exposure to acidic, alkaline, oil-based, and neutral food-contact environments. This study evaluates the chemical resistance, thermal cycling effects, and mechanical durability of thermochromic pigment–polymer blends. Thermochromic polymer samples were subjected to multiple chemical environments, repeated thermal cycling, and mechanical analysis to assess degradation behavior. The findings show that virgin food-grade polymer with no thermochromic pigment sustains its performance stability throughout chemical exposure with little degradation. However, thermochromic polymer blends experienced reduced thermochromic functionality. This study offers insight into how well thermochromic pigment can be incorporated into intelligent food packaging despite the limitations associated with chemical exposure.

## 1. Introduction

Assuring food safety and cutting down on waste are two challenges facing the food packaging industry today [1]. Unlike traditional passive materials, thermochromic pigments (TPs) actively indicate temperature fluctuations, offering a real-time, cost-effective solution for food safety monitoring [2]. However, their long-term stability in food-contact conditions remains uncertain. This study evaluates TP chemical resistance, thermal stability, and mechanical durability for food packaging, addressing critical gaps in their real-world performance [3].

Traditional food packaging protects food from damage and helps preserve them until they are ready for consumption [4]. Packaging also displays branding and nutritional information while supporting marketing efforts [5]. Ancient packaging included using wooden parts, especially bamboo baskets, wooden containers, and jute sacks, for packaging fruits and vegetables [6]. Over the past few decades, petroleum-based polymer-generated plastic films have been used as a barrier to protect food products from heat, moisture, microorganisms, dust, and dirt particles [7]. These plastic films are cost-effective, lightweight, and convenient materials with excellent barrier properties, resistance to stress and corrosion, and excellent transport and handling capabilities [8,9].

The market for consumables made from plastic has grown significantly [10]. A compound annual growth rate (CAGR) of 7.5% is anticipated for 2025. This would amount to $1621.51 billion in 2025, up from $1508.6 billion in 2023 [11]. In Europe, packaging represents the most prominent application of plastic [12]. Approximately half of all plastic products utilized in the food industry, including cutlery, plastic bags, coatings, and packaging materials, are designed for single-use disposal [13]. Over 35 million tons of waste from various plastic products are produced yearly; unfortunately, only 7% are recycled [14].

The increasing demand for functional packaging has driven interest in innovative technologies that respond to environmental stimuli and provide real-time food quality indicators [15]. Smart materials are advanced versions of conventional materials that can be used in many applications across various sectors [16]. These advanced materials are widely used in sensors, coatings, biomedical devices, and adaptive textiles, where environmental responsiveness is essential [17,18,19,20]. Chromogenic materials offer various applications across various sectors because they can undergo reversible or irreversible color changes in response to stimuli such as temperature (thermochromic), light (photochromic), or electric fields (electrochromic) [21]. Thermochromism refers to the phenomenon of color change exhibited by chromic materials in response to variations in temperature [22]. This process occurs due to molecular rearrangements, phase transitions, or electron transfer within the material, leading to visible shifts in color [23]. Thermochromic materials are commonly adopted in smart textiles, food packaging, security printing, and medical indicators, where temperature-sensitive visual indicators are beneficial [24,25,26,27].

Intelligent and active packaging are the two main categories of smart packaging [28]. Intelligent packaging detects changes in food quality within the package using indicators, sensors, and radio frequency identification (RFID) tags and communicates these changes through visual signals [29]. Active packaging extends a product’s shelf life by incorporating absorption and diffusion systems designed to regulate substances such as oxygen, carbon dioxide, and hydroalcoholic solution [30]. Intelligent packaging has become increasingly popular in the modern packaging sector due to its added value, enhanced safety features, and greater potential for marketing and promotional strategy [31]. Driven by growing consumer demands for sustainability, enhanced product safety, and high-quality standards in all areas of the life sciences, the food industry has increasingly adopted intelligent packaging solutions [32,33]. The first notable discovery of thermochromism was documented in 1888 when Friedrich Reinitzer identified the phenomenon in liquid crystals [34].

Thermochromic materials, which are used in a range of industrial applications from food packaging to textiles, can be divided into groups according to their chemical makeup and activation processes [35,36,37]. The main attractiveness of polymer-based thermochromic systems is their broad compatibility with flexible packaging materials [35].

Despite their potential, concerns regarding the functionality of innovative polymer materials have been highlighted in various applications [36]. In food packaging, thermochromic materials are valued for their precise temperature sensitivity [37]; however, concern regarding the effects of repeated thermal cycling on the immediate functionality of thermochromic polymers, particularly their ability to maintain consistent color-changing properties, is evident [38,39]. For TP to endure physical stresses in food packaging without degrading, mechanical integrity is essential, but little is known about how susceptible they are to these forces [40]. The aesthetic quality of TP food packaging requires further investigation, particularly regarding the resistance to surface blemishes [41,42].

Chemical sensitivity is a crucial factor in assessing the performance of TP in food-contact environments, as certain substances can alter polymer structure, affecting color change or degradation [43,44]. Thermochromic polymers used in food packaging are frequently exposed to various chemical agents during their lifecycle, including oils, acids, solvents, alkaline, and food-related substances [45]. Solutions may interact with packaging materials, potentially compromising their structural integrity, appearance, and functional properties and potentially affecting the functionality of thermochromic polymers [46]. Several studies have dealt with thermochromic inks’ low-light fastness issues [31,47,48]. However, research on the chemical resistance of thermochromic plastics in food packaging remains largely underexplored [49].

This study aims to evaluate thermochromic polymers’ chemical resistance, thermal cycling effects, and mechanical stability in simulated food-contact environments, addressing existing knowledge gaps.

The specific objectives of this study are as follows:

i. To assess thermochromic polymers’ optical stability and thermochromic activation before and after exposure to solutions representative of food-contact environments.
ii. To determine the impact of repeated thermal cycling on thermochromic behavior post-chemical exposure.
iii. To review mechanical integrity post-chemical exposure.

This research explores the feasibility of thermochromic polymers for commercial food packaging, seeking to develop durable, chemically stable, and reliable intelligent packaging solutions. By bridging the gap between thermochromic material development and real-world applications, manufacturers can optimize formulations for enhanced environmental resistance and integration into next-generation smart packaging solutions.

This study comprehensively assessed the chemical resistance, mechanical stability, and optical integrity of thermochromic polymers in food-contact situations, in contrast to earlier research that solely concentrated on the color responsiveness of thermochromic materials. It was the first study to integrate structural (SEM), mechanical (tensile, impact), and optical (color stability) analyses to assess real-world degradation mechanisms. The findings provide critical insights for the practical deployment of thermochromic intelligent packaging, especially regarding performance sustainability after chemical exposure.

## 2. Materials and Methods

### 2.1. Materials

Avenue Moulds Solutions Limited (Sligo, Ireland) provided a commercially available food-grade high-density polyethylene (HDPE) (Borealis MG7547S, Vienna, Austria). The material had a density of 0.954 g/cm^3^ and a melt flow rate (MFR) of 4 g/10 min (190 °C/2.16 kg load). Furthermore, the manufacturer verified the material as appropriate for food contact applications.

Pellets of TP (ThermoBatch^TM^) with a reversible temperature transition of 38 °C, were supplied by SpotSee^®^/LCR Hallcrest Ltd. (Chester, UK). The density was 0.508 g/cm^3^ with an MFI of 15–40 g/10 min. Unless stated otherwise, all testing was carried out at 23 °C ± 2 °C following ISO 527-2:2012 [50], ISO 179-1:2023 [51], and other applicable standards. Results from tensile, impact, and other property evaluations were ensured to be consistent and repeatable under these circumstances.

### 2.2. Blend Preparation

To achieve homogeneity, binary mixes of TP as the additive and HDPE as the matrix were manually dry-mixed by tumbling in a sealed polyethylene bag for five minutes. The weight of each blend was 2 kg. The components were precisely weighed using a Sartorius LA230P analytical balance with a precision of ±0.0001 g (Sartorius, Dublin, Ireland). A suitable polymeric carrier miscible with the HDPE matrix was present in the green-colored concentrate ThermoBatch^TM^. To guarantee consistent findings, the samples were conditioned at 23 °C for 24 h prior to testing after blending. Table 1 displays the compositions created for this study.

### 2.3. Injection Molding

Injection molding is a molding technique that uses an external heating device and a screw to melt the material, which is then injected into a mold to create the desired part while the mold cools [52]. Following ISO 294-1:2017 [53], injection molding was performed. An Arburg Allrounder 370E 600 E drive injection molding machine (Arburg, Lossburg, Germany) was used to process the blends, as shown in Table 1. Two tensile (type B1) and two impact (type A1) test specimens were produced by the machine using a “two by two” family mold with a double-T runner. The Arburg machine featured a maximum calculated stroke volume of 85 cm^3^, a maximum clamping force of 600 kN, and a screw diameter of 30 mm. 

Four temperature controllers controlled the temperature distribution along the barrel, while a fifth controller was used to control the temperature of the nozzle. Starting at 170 °C at the hopper, the temperature profile gradually rose to 210 °C at the nozzle. With an injection pressure of 750 bar, an injection speed of 80 mm/s, and a holding pressure of 400 bar, a cooling duration of 35 s was implemented. A Piovan Technologies THM 120/EN temperature control unit (JL Goor, Wicklow, Ireland) was used to keep the mold at a consistent 30 °C. The shot size of the mold was 52 g. For every blend formulation shown in Table 1, tensile test specimens (ISO 527-2:2012 specimen type 1BA [50]) were molded. Charpy impact test specimens were also created on the same mold and, when necessary, notched with a Type A V-notch (ISO 179-1:2023: specimen type A1, direction of blow edgewise [51]).

### 2.4. Chemical Resistance Testing Solutions

To evaluate the chemical resistance of the thermochromic polymer blends, each blend was exposed to a series of solutions representing different food-contact environments. The solutions were selected to simulate real-world exposure scenarios across a broad pH spectrum (2.3–12.5), including acidic, neutral, solvent-based, oil-based, and alkaline conditions. 

The pH of each solution was measured using an Orion Model 410a Lab Professional pH meter paired with a Hanna HI 1110B combination pH electrode (Thermofisher Scientific, Leicester, UK). Before testing, the pH meter was calibrated with standard buffer solutions at pH 4, 7, and 10. The measured pH values were recorded to ensure consistency, and periodic monitoring confirmed that no significant pH drift occurred during exposure.

The solutions tested represented a variety of food-contact conditions. Acidic conditions were simulated using lemon juice (pH 2.3) and orange juice (measured pH 3.8) to replicate highly acidic beverages and fruit-based products. As a baseline reference, neutral conditions were evaluated using tap water (pH 7.0). A 40% hydroalcoholic solution, prepared by diluting ethanol in deionized water (pH 7.2), was used to mimic solvent-based environments typical of alcohol-containing food products. Oil-based conditions were evaluated using vegetable oil (non-aqueous; no pH value) to assess interactions with fatty food components. Alkaline conditions were simulated using a 5% bleach solution (measured pH 12.5) prepared by diluting commercial bleach in deionized water to assess material resistance in highly alkaline environments.

Each thermochromic polymer blend (HDPE100/TP0, HDPE98/TP2, and HDPE92/TP8) was fully submerged in 3 L of the test solution for 24, 48, and 72 h to simulate both short-term and prolonged exposure. Due to vessel interactions, non-reactive Pyrex^®^ borosilicate glass containers prevented contamination or unintended pH fluctuations. Table 2 summarizes the test solutions, concentrations, measured pH values, and the exposure times.

Following exposure, the samples were gently blotted with lint-free absorbent paper and left to air dry under monitored temperature conditions (23 ± 2 °C) in a controlled laboratory environment for 24 h before subsequent analysis.

### 2.5. Visual and Spectrophotometric Color Analysis

Spectrophotometry is a method used to measure the interaction of light with materials [54]. The chromatic characteristics and color stability of thermochromic materials exposed to multiple solutions were examined using a portable, handheld sphere spectrophotometer (X-Rite SP62, Grand Rapids, MI, USA). The spectrophotometer was calibrated with a white tile and a zero tile in sequence before taking the measurements following the SP60 Series Spectrophotometer user instructions. The color results were presented using the Commission International de l’Eclairage (CIE) chromaticity coordinate system (L*, a*, b*) [55].

In this system, the L* value represents the luminance between black and white and ranges from 0 (black) to 100 (white) [56]. The a* and b* parameters represent chromaticity without specific numerical limits. Negative a* values correspond to green, while positive a* values correspond to red. Similarly, negative b* values correspond to blue and positive b* values to yellow [57]. The differences (ΔL*, Δa*, and Δb*) indicate the variation between the sample and standard in L*, a*, and b* values [58]. In addition, the total color difference (${\Delta E}_{ab}^{*}$) was calculated using Equation (1).

(1) $${\Delta E}_{ab}^{*}\;=\;[{\left(\Delta L^{*}\right)}^{2}+({\Delta a^{*})}^{2}+{\left(\Delta b^{*}\right)}^{2}{]}^{1/2}$$

where

${\Delta E}_{ab}^{*}$ is the color difference between two colors;

$\Delta L^{*}$ is the difference in lightness between the two colors;

$\Delta a^{*}$ is the difference in the red–green axis;

$\Delta b^{*}$ is the difference in the yellow–blue axis.

The color coordinates L*, a*, and b* were recorded according to ISO/CIE 11664-4:2019 [59] using Oncolor™ (v.6.3.4.4 QC-Lite) software. Five replicates were obtained for each group of samples, and the corresponding means and standard deviations were calculated. This coordinate made it possible to determine the color difference associated with the test specimens. The color difference, represented by the distance metric ∆E*_ab_, was obtained using Equation (1) by comparing the color coordinates of the chemically exposed samples with those of the unexposed food-grade material samples, which served as a reference.

The selected test solutions—lemon juice, orange juice, tap water, hydroalcoholic solution, vegetable oil, and bleach—were chosen to reflect a wide range of chemical exposures typically relevant to food-contact materials as recommended by regulatory standards. No additional chemicals were assessed beyond those listed. Future studies could expand chemical resistance evaluations to include organic solvents, enzymatic conditions, or industrial cleaning agents to broaden environmental relevance further.

### 2.6. SEM Analysis

Scanning electron microscopy (SEM) is a characterization technique for obtaining high-resolution surface topography of test specimens [60]. SEM is a technique that uses a focused electron beam to scan the surface of a specimen, enabling detailed morphological analysis in three dimensions [61].

Fractured injection-molded impact bars were analyzed using a Tescan Mira SEM (Oxford Instruments, Cambridge, UK) to examine external surface topography and internal fracture morphology. Before imaging, samples were affixed to adhesive conductive tape on aluminum stubs. A gold coating was applied using a Baltec SCD 005 sputter coater (BAL-TEC GmbH, Schalksmühle, Germany) to enhance surface conductivity and optical contrast.

SEM imaging was conducted at 100× magnification to assess surface defects, microcracks, and pigment distribution after subjecting samples to six test solutions. This study emphasized the effects of stress concentration and TP dispersion.

### 2.7. Moisture Content Analysis

Moisture content testing is crucial in various industries, including food, to ensure product quality, stability, safety, as well as prevent microbial growth, extend shelf life, and maintain material integrity [62,63,64]. In the food sector, controlling moisture is essential to prevent microbial growth, extend shelf life, and maintain material integrity. In this study, moisture content was measured to assess potential material degradation, hygroscopic behavior, and the impact of chemical exposure on thermochromic polymer blends.

A Rolbatch RBMB-210 Moisture Meter (Mettler-Toledo, Columbus, OH, USA) with a measurement resolution of ±0.001 g was used to determine the moisture content of test materials. Accurate evaluation of moisture absorption and retention was made possible by the moisture meter’s integrated halogen lamp, which was adjusted to a drying temperature of 120 °C.

To accommodate the moisture analyzer’s disposable pan, impact test specimens were granulated using a Rapid 150-21 series rotary granulator (Rapid Granulator, Bredaryd, Sweden). Granules weighing 4 g were placed into the moisture analyzer’s disposable pan.

Five replicate specimens were evaluated for each solution to produce statistically significant results.

### 2.8. Mass Stability

Mass measurements taken prior to testing specimens demonstrated that the mechanical properties were consistent and comparable between tests [65]. To guarantee the comparability of mechanical properties across various solutions, the mass of five replicate tensile test specimens was determined using a Mettler TG50 Thermobalance (Mettler-Toledo, Columbus, OH, USA), with a precision of ±0.0001 mg. These analyses helped to identify potential mass variations resulting from material degradation, leaching, or uptake of solution components during chemical exposure [66,67,68].

### 2.9. Thermal Cycling

Depending on their chemical makeup, formulation, and intended use, thermochromic polymers exhibit a wide range of transition temperature ranges [69]. Their thermal and optical characteristics were assessed before and after chemical exposure using temperature-controlled reflectance spectroscopy. The experimental setup included a digitally controlled temperature-regulated hotplate (Fisherbrand™ AREX 5, ±0.5 °C accuracy) with a Pt100 external temperature probe (Fisher Scientific Ireland Limited, Dublin, Ireland) to ensure precise and uniform heating and minimize temperature fluctuations that could affect thermochromic activation.

A handheld portable UV–Vis spectrophotometer (Konica Minolta CM-700d, Konica Minolta, Warrington, UK) equipped with an integrating sphere was used to quantify reflectance changes as a function of temperature. Color stability testing was conducted on HDPE100/TP0, HDPE98/TP2, and HDPE92/TP8 tensile specimens before and after chemical exposure to assess whether chemical interactions altered thermochromic activation behavior.

To simulate real-world temperature fluctuations, specimens underwent 100 cyclic heating–cooling cycles. In each cycle, the temperature was raised from 23 °C to 50 °C degrees Celsius and then passively cooled to room temperature. Each specimen’s surface temperature was tracked in real time by a thermocouple placed in the middle of the sample.

Potential changes in activation temperature brought on by chemical exposure and repetitive thermal cycling were assessed using the manufacturer’s stated transition temperature (38 °C) as a guide. Surface temperatures were kept between 33 °C and 43 °C throughout the spectrophotometric examination to guarantee thorough and uniform color changes in every studied specimen. This range was selected to capture the start and finish of the thermochromic reaction while including the whole activation window and avoiding overheating that could result in deterioration.

Spectroscopic reflectance measurements were recorded at regular temperature intervals to quantify variations in optical properties during activation. A visual assessment was conducted before, during, and after thermal cycling to detect potential fading, irreversible color shifts, or degradation in pigment activation.

The thermal cycling procedure was designed to replicate common temperature fluctuations encountered during the lifecycle of food packaging, such as movement between cold storage and ambient environments or end-user handling. Samples underwent 100 cycles, each consisting of heating from 23 °C to 50 °C (±0.5 °C), followed by passive cooling to room temperature. This range captured the thermochromic pigment’s full activation window (33–43 °C) and mimicked operational temperature shifts without exceeding degradation thresholds. The number of cycles reflected multiple use exposures relevant to intelligent packaging scenarios like cold chain logistics, product display, or consumer handling.

### 2.10. Tensile Testing

Tensile testing of polymers is used to evaluate a polymer’s mechanical properties by applying a controlled tensile (pulling) force until the sample breaks [56]. Tensile testing was performed on tensile specimens (specimen type 1BA) per ISO 527-2:2012 [53] to evaluate the mechanical properties of the polymer blends post-chemical exposure. To evaluate the ductility and toughness of the samples, the analysis examined the maximum tensile stress, Young’s modulus, and tensile strain at break. In order to achieve statistical robustness, five replicate specimens per cycle were used to test each polymer blend, HDPE100/TP0, HDPE98/TP2, and HDPE92/TP8, which had been exposed to the chemical solutions.

The tensile test was conducted using an Instron 3400 tensile tester machine (Instron, Norwood, MA, USA), configured with a 4 kN load cell and Bluehill^®^ software version 4.29 for data gathering. Five specimens, each measuring 170 mm in length, 10 ± 0.2 mm in width, and 4 ± 0.2 mm in thickness, were used to examine each sample. A grip gap of 25.4 mm was maintained to guarantee that the clamping of the samples was even. Measurement testing was carried out using a Mitutoyo Absolute CD-6-ASX caliper (BCS Calibration, Laois, Ireland). Tensile testing was performed at a 10 mm/min strain rate in steady-state conditions.

### 2.11. Charpy Impact Testing

The Charpy test is a standardized method for assessing a material’s impact strength and toughness [70]. A pendulum hammer is released to strike the specimen, and the resulting impact energy is recorded [71]. Charpy impact testing was conducted on ten specimens following ISO 179-1:2023 [51] using a calibrated CEAST Resil 6545 5.5 Series pendulum impact testing machine (Zwick Roell, Ulm, Germany). Notched and unnotched specimens were tested to evaluate the effect of chemical exposure on impact resistance.

Test specimens, with an average thickness of 12.72 mm (±0.04 mm), were prepared with a Type A V-shaped notch (2 mm depth) using a Zwick/Roell notch cutter (Zwick/Roell, Ulm, Germany). Impact testing was conducted using a 4 J hammer mounted on a swinging pendulum operating at an impact velocity of 2.9 m/s. Each specimen was horizontally centered, ensuring optimal notch alignment with the pendulum arm and positioning away from the impact direction.

The hammer arm was released from an elevated position and struck the specimen. Notched specimens fractured upon impact, whereas unnotched specimens remained intact. The subsequent downward motion of the weighted pendulum enabled the determination of the specimen’s impact energy in joules.

The impact absorption energy of each sample was obtained from the impact tester display. The corresponding Charpy impact strength, *a_cU_*, expressed in kilojoules per square meter (kJ/m^2^), was calculated for unnotched samples using Equation (2):

(2) $$a_{cU}=\frac{Wc}{h\times b}\times 10^{3}$$

where

*Wc* is the corrected energy, in joules, absorbed by breaking the test specimen;

*h* is the thickness, in millimeters, of the test specimen;

*b* is the width, in millimeters, of the test specimen.

The Charpy impact strength, *a_cN_*, expressed in kilojoules per square meter (kJ/m^2^), was determined for the notched samples using the calculation presented in Equation (3):

(3) $$a_{cN}=\frac{Wc}{h\times b_{N}}\times 10^{3}$$

where

*Wc* is the corrected energy, in joules, absorbed by breaking the test specimen;

*h* is the thickness, in millimeters, of the test specimen;

*b_N_* is the width, in millimeters, of the test specimen.

### 2.12. Statistical Analyses

For any research activity to reach quantitative conclusions, statistical analysis is necessary [72]. Quantitative analysis is made more rigorous and reliable using systematic and consistent data collection. All data points were kept in the dataset, and no outliers were removed. The sample size for each test was explicitly defined to ensure methodological transparency and reproducibility. Statistical analyses were performed using Minitab^®^ 21.4.1 Statistical Software. Normality testing was carried out on each set of results to see whether to accept or reject the hypothesis and if there was a statistical difference in the results of the samples post-chemical resistance testing. A one-way analysis of variance (ANOVA) was performed for quintuplicate (*n* = 5) and decuplicate (*n* = 10) measurements, with results expressed as mean  ±  standard deviation. Post hoc comparisons were conducted using the Tukey method, and statistical significance was determined at *p* < 0.05, with a 95% confidence level. The conclusions considered statistical significance and practical relevance to ensure meaningful interpretation of the data.

In addition, measurement uncertainties associated with mass (±0.0001 mg), tensile testing (±1% load cell accuracy), thermal cycling (±0.5 °C), moisture analysis (±0.001 g), and colorimetric analysis (${\Delta E}_{ab}^{*}$ ± 0.2) were considered. Results are reported as mean ± standard deviation to account for natural sample variability.

### 2.13. Rationale for Testing Conditions

The selection of chemical exposure solutions, exposure durations, thermal cycling parameters, and mechanical testing protocols was guided by the need to simulate realistic food-contact and packaging lifecycle conditions. Solutions representing acidic (lemon juice, orange juice), neutral (tap water), solvent-based (hydroalcoholic solution), oil-based (vegetable oil), and alkaline (bleach solution) environments were chosen to reflect common foodstuffs and cleaning agents. Exposure durations of 24, 48, and 72 h allowed the assessment of short- and moderate-term effects on material stability. Thermal cycling between 23 °C and 50 °C was selected to replicate temperature fluctuations typical of food logistics and storage, focusing on the thermochromic activation temperature range (33–43 °C). Tensile and impact testing followed internationally recognized ISO standards to ensure results were relevant to packaging performance evaluations.

## 3. Results

### 3.1. Visual Appearance and Colorimetric Results

**Figure 1:**
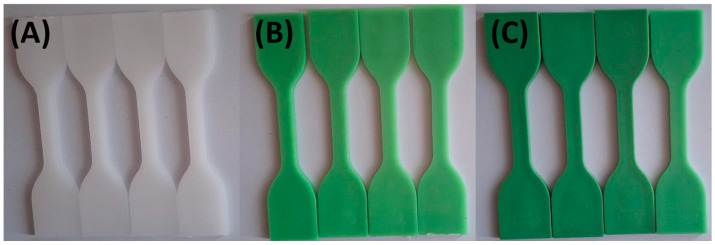
Visual color changes in (**A**) HDPE100/TP0, (**B**) HDPE98/TP2, and (**C**) HDPE92/TP8 before and after exposure to lemon juice. Each set shows samples in the following order: unexposed (control), 24 h, 48 h, and 72 h. Images are representative of five replicate specimens per condition (*n* = 5).

**Figure 2:**
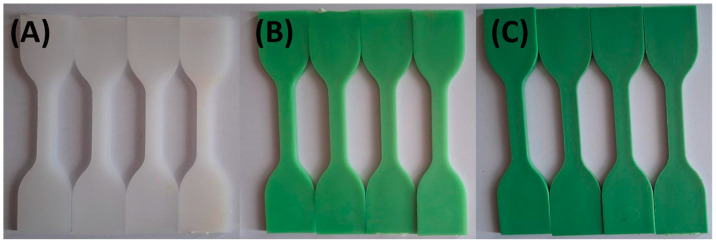
Visual color changes in (**A**) HDPE100/TP0, (**B**) HDPE98/TP2, and (**C**) HDPE92/TP8 before and after exposure to orange juice. Each set shows samples in the following order: unexposed (control), 24 h, 48 h, and 72 h. Images are representative of five replicate specimens per condition (*n* = 5).

Photographs were taken after samples had been exposed to the various test solutions at 0, 24, 48, and 72 h to document color changes. Figure 1 and Figure 2 show that specimens exposed to lemon and orange juice exhibited progressive discoloration over time. By 72 h, HDPE100/TP0 (white) showed minimal change, whereas HDPE98/TP2 and HDPE92/TP8 (green) displayed increasing lightening, with HDPE98/TP2 exhibiting the most significant shift. The loss of green hue suggests prolonged acid exposure degrades TP, indicating potential limitations for food packaging applications.

**Figure 3:**
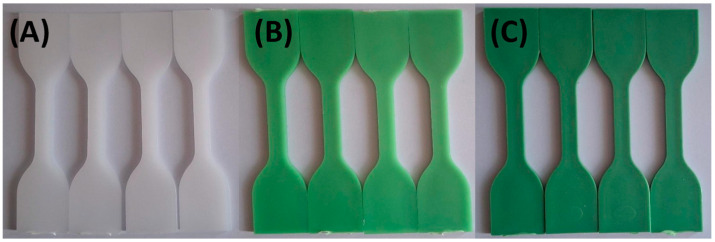
Visual color changes in (**A**) HDPE100/TP0, (**B**) HDPE98/TP2, and (**C**) HDPE92/TP8 before and after exposure to tap water. Each set shows samples in the following order: unexposed (control), 24 h, 48 h, and 72 h. Images are representative of five replicate specimens per condition (*n* = 5).

**Figure 4:**
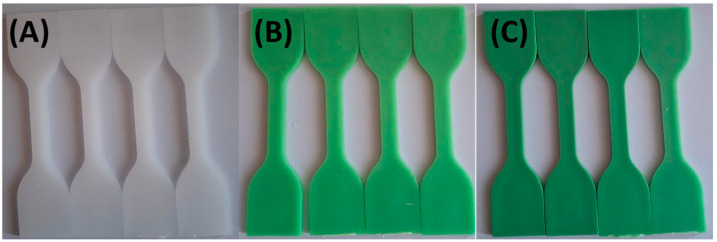
Visual color changes in (**A**) HDPE100/TP0, (**B**) HDPE98/TP2, and (**C**) HDPE92/TP8 before and after exposure hydroalcoholic solution. Each set shows samples in the following order: unexposed (control), 24 h, 48 h, and 72 h. Images are representative of five replicate specimens per condition (*n* = 5).

Figure 3 and Figure 4 illustrate the limited impact of tap water and hydroalcoholic solution on color stability. Throughout the 72 h exposure, only minimal color change was observed in any formulation, suggesting that these solutions interact minimally with the polymer matrix and TP.

**Figure 5:**
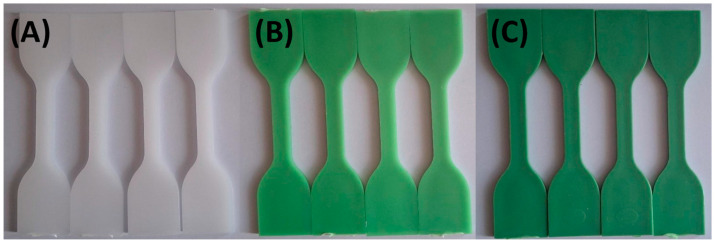
Visual color changes in (**A**) HDPE100/TP0, (**B**) HDPE98/TP2, and (**C**) HDPE92/TP8 before and after exposure to vegetable oil. Each set shows samples in the following order: unexposed (control), 24 h, 48 h, and 72 h. Images are representative of five replicate specimens per condition (*n* = 5).

As shown in Figure 5, vegetable oil exposure resulted in slight fading of green shades in HDPE98/TP2 and HDPE92/TP8 by 72 h. However, this effect was less pronounced than that observed with acidic solutions. The fading may be attributed to surface interactions [73], oil absorption [74], or changes in light reflection rather than pigment degradation [75].

**Figure 6:**
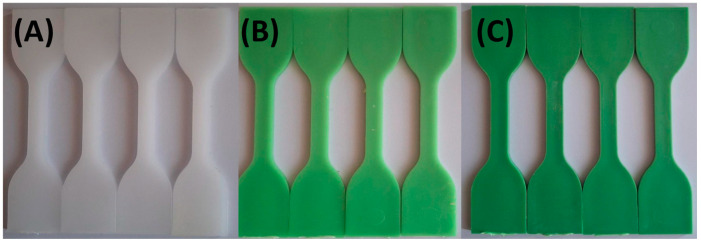
Visual color changes in (**A**) HDPE100/TP0, (**B**) HDPE98/TP2, and (**C**) HDPE92/TP8 before and after exposure to bleach solution. Each set shows samples in the following order: unexposed (control), 24 h, 48 h, and 72 h. Images are representative of five replicate specimens per condition (*n* = 5).

Figure 6 demonstrates the most pronounced color fading in HDPE98/TP2 and HDPE92/TP8 after bleach solution exposure. By 72 h, the green color had noticeably faded, indicating that bleach interacts with TP or the polymer matrix, leading to chemical oxidation or pigment breakdown [76]. HDPE100/TP0 showed little to no visible change, likely due to the absence of colorants in its formulation. The results confirm that oxidizing agents accelerate pigment degradation, affecting long-term material stability [77].

Table 3 presents the total color difference (${\Delta E}_{ab}^{*}$) for thermochromic polymer specimens subjected to different test solutions. The ${\Delta E}_{ab}^{*}$ values in Table 3 support the visual observations. HDPE100/TP0 exhibited minimal changes (${\Delta E}_{ab}^{*}$ 0.11–1.89), while HDPE98/TP2 and HDPE92/TP8 displayed more noticeable color shifts (${\Delta E}_{ab}^{*}$ 0.31–3.75), especially in response to acidic and oxidizing solutions. A one-way ANOVA was performed on ${\Delta E}_{ab}^{*}$ values at 24, 48, and 72 h to assess whether the observed color differences over time were statistically significant within each solution and specimen group. The findings confirmed that extended exposure increases pigment degradation by showing substantial differences (*p* < 0.05) in HDPE92/TP8 samples exposed to acidic and oxidizing solutions, specifically HDPE92/TP8 in orange juice, bleach, and lemon juice.

The extent of discoloration and ∆E*_ab_ values increased with pigment concentration. HDPE92/TP8 consistently exhibited the most pronounced color change across acidic and oxidative solutions, suggesting that higher pigment loading increases susceptibility to chemical degradation.

The degradation of thermochromic functionality in acidic environments is likely driven by two mechanisms. Firstly, acidic solutions may protonate or hydrolyze components of the microencapsulated thermochromic system, disrupting the color change equilibrium between the leuco dye and color developer [78]. Secondly, acid exposure may compromise the encapsulation or the polymer matrix itself, as evidenced by the observed surface microcracks and increased roughness in SEM images [79]. These physical changes may promote pigment leaching, moisture penetration, and reduced chromophore stability, contributing to irreversible discoloration or reduced activation efficiency.

### 3.2. SEM Observations

SEM examination of the injection-molded bars’ fracture morphology and surface topography was conducted at 100× after exposure to various test substances for 0, 24, 48, and 72 h. SEM analyses revealed minimal microstructural degradation in HDPE98/TP2 and HDPE92/TP8, while HDPE100/TP0 exhibited relatively stable morphology across all exposure conditions and time points.

**Figure 7:**
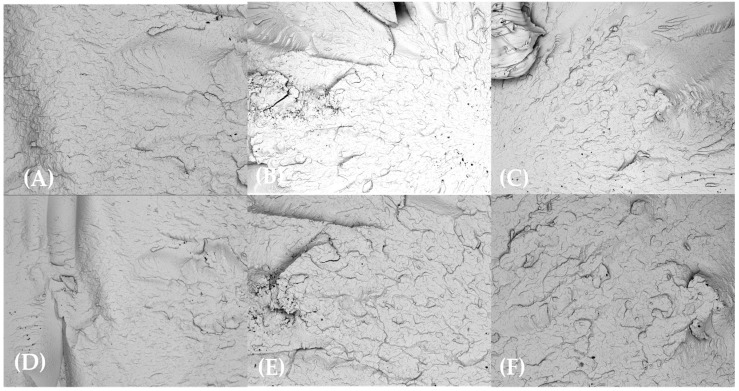
SEM micrographs of HDPE100/TP0 post-72 h exposure at 100× magnification: (**A**) lemon juice, (**B**) orange juice, (**C**) tap water, (**D**) hydroalcoholic solution, (**E**) vegetable oil, and (**F**) bleach solution.

As shown in Figure 7, HDPE100/TP0 exhibited minimal signs of degradation across all test substances exposures at 100× magnification. In applications with longer exposure, the virgin polymer blend’s structural stability was confirmed by the smooth fracture surfaces that showed no discernible microcracks or pigment clustering. Given its constant morphology, HDPE100/TP0 is a suitable choice for high-durability applications, suggesting that it maintains its mechanical integrity after exposure to various test substances.

**Figure 8:**
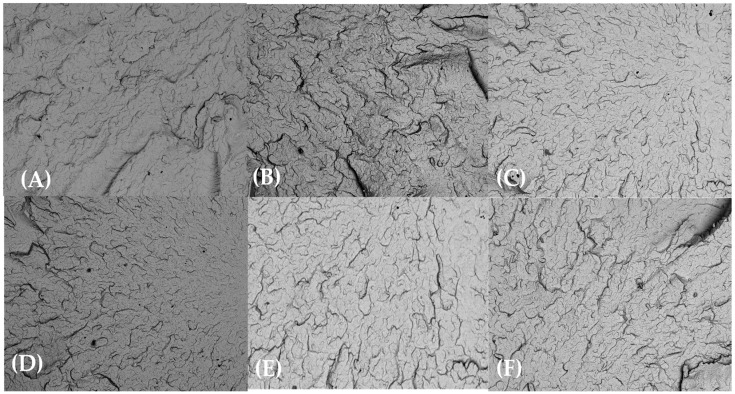
SEM micrographs of HDPE98/TP2 post-72 h exposure at 100× magnification: (**A**) lemon juice, (**B**) orange juice, (**C**) tap water, (**D**) hydroalcoholic solution, (**E**) vegetable oil, and (**F**) bleach solution.

In contrast, HDPE98/TP2 (Figure 8) displayed moderate degradation, with noticeable surface roughness and pigment clustering emerging after chemical exposure. Localized microcracks and roughened fracture edges were observed in orange juice, lemon juice, and bleach solution test specimens, suggesting early mechanical weakening.

**Figure 9:**
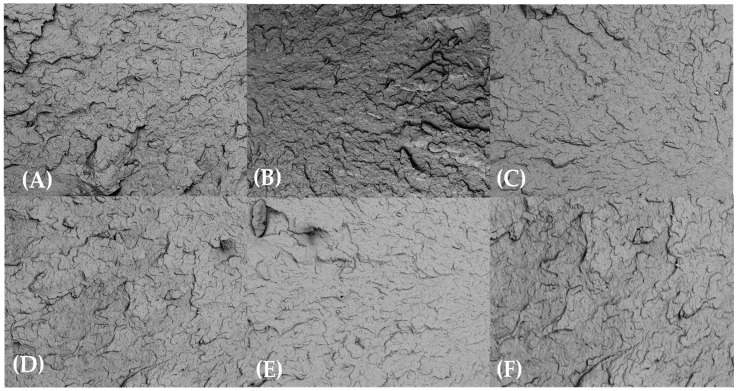
SEM micrographs of HDPE92/TP8 post-72 h exposure at 100× magnification: (**A**) lemon juice, (**B**) orange juice, (**C**) tap water, (**D**) hydroalcoholic solution, (**E**) vegetable oil, and (**F**) bleach solution.

The most severe degradation was seen in HDPE92/TP8 (Figure 9), which showed widespread cracking, pigment grouping, and roughness with the acidic and oxidizing agent environments. This indicates that higher pigment content not only increases thermochromic intensity but also introduces morphological weaknesses under chemical stress.

Brittle failure and mechanical instability resulted from the uneven dispersion of TP acting as stress concentration spots, speeding up the initiation and spread of cracks [80].

While the material remains suitable for limited-use applications, such as dry food packaging, outer packaging layers, and tamper-evident seals and caps, its potential embrittlement during food-contact chemical environments may reduce its long-term structural performance.

### 3.3. Moisture Content Analysis Results

Table 4 summarizes the moisture content results for the polymer blends (HDPE100/TP0, HDPE98/TP2, and HDPE92/TP8) before and after exposure to test substances. The moisture content values represent the mean of five replicate specimens per test solution exposure.

The control samples (HDPE100/TP0) showed low inherent moisture absorption while maintaining a constant moisture content. These moisture content findings support HDPE’s hydrophobic properties, which prevent water absorption in typical circumstances.

Acidic solutions (lemon and orange juice) increased moisture content over time, with HDPE98/TP2 and HDPE92/TP8 absorbing more than HDPE100/TP0, likely due to acid-induced surface interactions, minor swelling, or increased roughness [81]. This enhanced moisture absorption was attributed to acid-induced surface degradation of the polymer matrix, as supported by SEM observations. Acid exposure likely created microcracks and surface irregularities, increasing hydrophilic surface area and facilitating water uptake. In contrast, exposure to neutral (tap water), solvent-based (hydroalcoholic solution), and oil-based (vegetable oil) environments resulted in comparatively lower moisture absorption, consistent with the inherent hydrophobicity of HDPE and the limited polymer–chemical interaction in less aggressive media. Tap water caused a moderate rise in moisture content, lower than acidic solutions, reinforcing the more significant permeability effect of acids [82]. Due to its low polarity, hydroalcoholic solution produced very little change in moisture content [83]. Vegetable oil showed little absorption, proving that non-polar substances do not help hydrophobic polymers to absorb moisture [84]. Bleach solution increased moisture, especially in HDPE98/TP2 and HDPE92/TP8, possibly because of oxidative effects, although not as much as in acidic solutions. Materials’ surface properties can be altered by oxidation, which may increase the materials’ affinity for water and increase their capacity to absorb moisture [85]. While HDPE98/TP2 and HDPE92/TP8 showed somewhat higher absorption, HDPE100/TP0 retained the least moisture throughout all exposures, indicating that thermochromic masterbatch may have a minimal impact on moisture interaction.

The higher moisture absorption observed in HDPE98/TP2 and HDPE92/TP8, compared to HDPE100/TP0, is attributed to thermochromic pigment particles, which introduced structural discontinuities into the HDPE matrix. These microstructural imperfections, combined with acid- and oxidant-induced surface degradation, increased the susceptibility to moisture uptake. Over time, exposure to acidic and bleach solutions progressively enhanced the hydrophilicity of the blends, as indicated by the rising moisture content measurements across 24, 48, and 72 h.

A one-way ANOVA revealed a statistically significant effect of chemical exposure on the moisture content of HDPE92/TP8 after 72 h. Tukey’s test indicated that samples exposed to orange juice, lemon juice, and bleach solution had significantly higher moisture uptake than the control, tap water, hydroalcoholic solution, and vegetable oil. These findings suggest acidic environments promote greater water absorption, likely due to polymer matrix interaction or surface degradation.

Over the 72 h exposure period, acidic environments (lemon and orange juice) induced the most pronounced moisture absorption, particularly in HDPE92/TP8, where the content increased from 0.04% to 0.22%. This suggests acid-induced matrix softening or surface erosion, which increased permeability. Bleach exposure resulted in a moderate moisture increase, likely from surface oxidation enhancing hydrophilicity. Tap water caused a gradual rise in moisture but to a lesser extent. In contrast, the hydroalcoholic solution and vegetable oil caused minimal changes, reflecting limited moisture affinity due to their non-polar or low-polarity composition. These results reinforced the conclusion that both chemical nature and exposure time significantly influence moisture interaction in thermochromic systems.

### 3.4. Mass Stability Results

Table 5 summarizes the mass measurements of tensile test specimens for the polymer blends (HDPE100/TP0, HDPE98/TP2, and HDPE92/TP8) across six test solutions. The mass values represent the mean of five replicate specimens per test solution.

The control samples for all three specimens maintained a consistent mass of 3.724 g, demonstrating no measurable weight change under standard conditions. Exposure to lemon juice, orange juice, and bleach solution resulted in a slight but noticeable increase in mass over time for HDPE98/TP2 and HDPE92/TP8, while HDPE100/TP0 showed less pronounced changes but was still present. This increase suggests that the acidic environment contributed to slight surface absorption or minor interactions with the TP, particularly in specimens containing higher pigment concentrations. The increase in mass aligns with moisture uptake results, reinforcing the idea that acidic solutions interact with the polymer matrix to a small extent.

Tap water showed modest mass gain, while hydroalcoholic solution and vegetable oil had negligible effects, reinforcing HDPE’s hydrophobic nature. The stability of HDPE100/TP0 suggests minimal interaction with chemicals, while higher TP concentrations in HDPE98/TP2 and HDPE92/TP8 led to more significant variations. These trends align with moisture uptake data, confirming that acids and bleach solution induce the most noticeable changes, while hydroalcoholic solution and oil have minimal impact.

All specimens showed total mass increases, indicating that these thermochromic polymer blends maintain stability when exposed to different chemical environments. These findings point to possible appropriateness for uses where a small amount of mass loss is acceptable and exposure to chemical solutions is necessary. Both moisture and mass uptake were highest in HDPE92/TP8 across most test solutions, especially acids. This pointed to greater permeability or polymer–pigment interaction at elevated pigment concentrations.

A one-way ANOVA showed a statistically significant effect of chemical exposure on the mass of HDPE92/TP8 after 72 h (*p* < 0.05). Tukey’s post hoc test indicated that samples exposed to orange juice, lemon juice, and bleach solution had significantly higher mass than the control, suggesting uptake due to surface interaction or fluid retention. In contrast, samples exposed to hydroalcoholic solution, tap water, and vegetable oil showed no significant difference from the control, indicating limited mass transfer or polymer interaction in non-polar or neutral environments. No significant differences were observed across polymer formulations, indicating that pigment concentration alone did not affect the extent of mass change. These findings suggest that environmental chemistry, rather than blend composition, primarily drives surface-level interactions that influence absorption.

The observed mass and surface integrity changes under acidic and oxidative conditions implied the possibility of low-level diffusion or degradation, even though this work lacked detailed migration or leaching investigations. These results highlighted the significance of conducting additional research on possible chemical release or interactions with food simulants, particularly in cases of extended exposure.

### 3.5. Effect of Thermal Cycling

Table 6 presents the effect of one hundred thermal cycles on the activation temperature of three polymer blends (HDPE100/TP0, HDPE98/TP2, and HDPE92/TP8) before and after exposure to various test solutions.

Chemical exposure impacts the activation temperature of thermochromic polymers to differing degrees, depending on the type of solution (see Table 6). All control samples maintained their stability at 38.1 °C across all formulations, demonstrating the dependability of the thermochromic activation temperature.

Because acidic solutions such as orange juice break down the pigment [86] or polymer matrix [87], they generate the most noticeable drop in activation temperature (−4.2 °C). According to the idea that neutral aqueous solutions have little effect on the stability of polymers, tap water had little effect on HDPE92/TP8, causing only a −0.7 °C change [88].

After hydroalcoholic solution exposure, the activation temperature shifted (−0.9 °C), suggesting that minor polymer–pigment interactions or solvation effects may impact pigment performance. Since vegetable oil is non-polar, it has a more negligible influence than an acidic solution (−1.3 °C).

Bleach solution exposure caused a significant decrease in activation temperature (−2.4 °C), indicating oxidative degradation of TP. This noteworthy decrease confirms earlier research that bleach solution increases pigment deterioration, which may change transition kinetics and lessen thermochromic response stability.

Similar patterns were shown by HDPE98/TP2 and HDPE92/TP8 across all formulations, confirming that the thermochromic masterbatch—rather than the polymer composition—is the main element affecting reaction changes. The information demonstrates that whereas neutral and non-polar compounds cause only minor changes in thermal stability, aggressive chemicals (such as lemon and orange juice) have a major impact.

### 3.6. Tensile Properties

Table 7 summarizes the tensile properties of HDPE100/TP0 over 0, 24, 48, and 72 h. The analysis considers tensile strain at break, maximum tensile stress, and Young’s modulus, providing insight into the material’s structural response under different conditions.

HDPE100/TP0 maintained stable tensile properties across most chemical exposures, with water, hydroalcoholic solution, and vegetable oil causing minimal changes. Lemon and orange juice initially reduced the tensile strain and Young’s modulus but showed recovery over time, suggesting minor polymer interactions. Bleach solution exposure resulted in progressive degradation, with the most significant drop in Young’s modulus (694.36 MPa at 72 h), indicating a loss of stiffness and potential plasticization of the matrix. HDPE100/TP0 remained mechanically robust, but acidic and oxidative environments may impact long-term performance in food packaging applications.

HDPE98/TP2 (Table 8) exhibited moderate variations in tensile properties across different chemical exposures over 72 h. Lemon and orange juice initially increased tensile strain and Young’s modulus, but a decline at 72 h suggested potential acid-induced polymer relaxation. Tap water and hydroalcoholic solution exposure reduced tensile strain, indicating minor softening, while maximum stress remained stable. Vegetable oil caused minor fluctuations, suggesting limited polymer interaction. Bleach solution exposure initially increased tensile strain but led to irregular trends in mechanical properties, possibly due to oxidative effects.

HDPE92/TP8 (Table 9) showed notable variations in tensile properties across different chemical exposures over 72 h. Lemon and orange juice caused fluctuations in tensile strain and Young’s modulus, suggesting acid-induced polymer interactions, with initial increases followed by declines. Tap water maintained stability, with only minor variations in Young’s modulus and tensile strain. Hydroalcoholic solution exposure resulted in inconsistent trends, with an early drop in Young’s modulus but recovery by 72 h, indicating potential plasticization effects. Vegetable oil exposure led to minimal changes, while bleach solution exposure progressively reduced tensile properties, suggesting oxidative degradation. HDPE92/TP8 remained structurally stable but exhibited greater susceptibility to acid and oxidative conditions than HDPE100/TP0 and HDPE98/TP2.

These results highlight a strong correlation between chemical exposure and variations in stability and resistance to degradation. Acidic and oxidative exposures induced polymer interactions that, in some cases, progressively reduced tensile properties [89,90].

HDPE100/TP0 demonstrated the highest chemical resilience, maintaining consistent impact strength and tensile properties across all test conditions, with minimal structural changes over time. HDPE98/TP2 exhibited moderate stability, with minor reductions in tensile strain and Young’s modulus in acidic and oxidative environments, indicating some degree of polymer interaction. HDPE92/TP8 showed the most pronounced variations, particularly under acidic and oxidative exposure, where progressive reductions in Young’s modulus and tensile strain suggest greater susceptibility to chemical-induced degradation. These findings indicate that HDPE100/TP0 is the most suitable for long-term applications in chemically exposed environments. At the same time, HDPE98/TP2 and HDPE92/TP8 may require further stabilization in acidic or oxidative conditions, particularly in food packaging applications.

In direct comparison, HDPE100/TP0 maintained consistent mechanical performance with negligible variation in tensile strength, modulus, and strain across acidic (lemon and orange juice) and oxidative (bleach) exposures over 72 h. HDPE98/TP2 displayed minor reductions in mechanical stability, with slight declines in Young’s modulus and strain at break becoming more evident after prolonged acid and bleach exposure. However, properties generally remained within an acceptable range for functional use. In contrast, HDPE92/TP8 showed the most significant mechanical degradation under these aggressive conditions, with noticeable reductions in modulus and elongation, consistent with the microstructural weakening observed through SEM imaging. These results reinforce the trend that increasing thermochromic pigment concentration compromised mechanical resistance, particularly when subjected to chemically aggressive environments over time.

Across all tensile testing metrics (tensile strain at break, maximum tensile stress, and Young’s modulus), a one-way ANOVA and Tukey HSD analysis showed no statistically significant differences (*p* > 0.05) between polymer types or chemical exposures at 72 h. Despite minor variations observed descriptively—particularly in acidic and oxidative environments—the statistical results indicate that these changes are insignificant. This suggests that all three polymer blends (HDPE100/TP0, HDPE98/TP2, HDPE92/TP8) maintained consistent mechanical performance after short-term chemical exposure, reinforcing their suitability for applications requiring short-term structural stability.

These findings collectively indicate that adding thermochromic pigments compromises mechanical properties compared to standard food-grade HDPE. While the virgin HDPE (HDPE100/TP0) maintained consistent tensile and modulus values across all chemical exposures, the TP-modified blends showed more pronounced degradation in acidic and oxidative environments. This divergence in performance was especially noticeable in HDPE92/TP8, where pigment-induced stress concentration likely contributed to reduced structural integrity.

### 3.7. Charpy Impact Strength Results

Using both notched and unnotched samples, the impact strength of different polymer blends was assessed with exposure to various chemical food-contact conditions. The results in Table 10 demonstrate that HDPE100/TP0 maintained stable impact strength across all chemical exposures over 72 h, with unnotched values remaining consistently above 99.2 kJ/m^2^ and unnotched values remaining consistently above 23.1 kJ/m^2^. Minor variations observed in lemon and orange juice suggest only surface-level interactions rather than structural degradation. However, the impact strength of hydroalcoholic solution was marginally increased, potentially due to plasticization effects, while tap water and hydroalcoholic solution exposure did not exhibit any significant effects. The high chemical resistance of HDPE100/TP0 was further reinforced by the negligible changes that occurred as a consequence of exposure to vegetable oil and bleach solution. Bleach solution-exposed samples stabilized at 99.80 kJ/m^2^.

For HDPE98/TP2, the results (Table 11) show slightly more significant variations in impact strength over 72 h compared to HDPE100/TP0. While the initial values remained high, there was a noticeable reduction in impact strength after prolonged exposure to acidic solutions, mainly orange juice, where values decreased over time. This reduction suggests some interaction between the TP and the acidic environment, potentially affecting the polymer’s structural integrity. While all formulations maintained high-impact values, HDPE98/TP2 and HDPE92/TP8 showed more significant declines under acidic exposure than HDPE100/TP0. In contrast, neutral and non-polar chemicals caused negligible change in all cases.

HDPE92/TP8 (Table 12) exhibited the most significant reduction in impact strength following chemical exposure, particularly in lemon and orange juice, suggesting acid-induced pigment degradation. This effect correlated with its higher TP concentration, indicating that increased pigment loading may accelerate structural changes in acidic environments. Water and hydroalcoholic solution caused minor fluctuations without significant degradation, while vegetable oil and bleach solution maintained stable impact strength. The observed weakening in acidic solutions suggests the potential limitations of prolonged exposure to acidic food products, as higher pigment concentrations may compromise mechanical stability.

These findings suggest that HDPE100/TP0 offers the most remarkable chemical stability, while HDPE98/TP2 and HDPE92/TP8 show increasing susceptibility to acidic degradation, particularly in higher pigment concentrations. This study highlights the trade-off between thermochromic functionality and long-term mechanical durability, an important consideration for food packaging and chemical-exposed applications.

One-way ANOVA results revealed significant differences in unnotched and notched impact strength across the three polymer blends. HDPE100/TP0 consistently demonstrated the highest mechanical performance, with significantly greater unnotched and notched impact values than HDPE92/TP8. HDPE98/TP2 showed intermediate behavior, closely aligning with HDPE100/TP0 in notched tests but showing a slight reduction in unnotched strength. The decline in impact resistance with increasing pigment concentration—particularly under notched conditions—suggests that higher thermochromic loading may contribute to reduced fracture toughness, likely due to pigment-induced microstructural changes.

Impact strength values for TP blends were consistently lower than for the control HDPE100/TP0 across all solutions. Thermochromic pigment, particularly at higher loadings, introduced localized stiffness mismatches and potential crack initiation sites, thereby reducing toughness. This suggested a trade-off between added functionality and impact resilience.

Impact resistance followed a similar trend of HDPE100/TP0 > HDPE98/TP2 > HDPE92/TP8. The greater pigment loading in HDPE92/TP8 appeared to contribute to brittleness, especially after exposure to lemon juice, orange juice, and bleach solution.

### 3.8. Recommendations for Improving Chemical Resistance

Based on the observed degradation behavior under chemical exposure, several strategies can be proposed to enhance thermochromic polymer systems’ chemical resistance and durability. These include improving pigment microencapsulation to limit direct contact with aggressive environments, incorporating multilayer packaging designs with protective barrier layers, and using compatibilizers to enhance pigment dispersion and matrix adhesion. Long-term performance could also be significantly enhanced by choosing pigments with greater intrinsic chemical resistance and adding stabilizers effective against oxidative and acid degradation. Future studies should investigate these strategies to maximize the stability and useful life of thermochromic materials in applications involving intelligent food packaging.

## 4. Conclusions

The chemical resistance, thermal stability, and mechanical durability of thermochromic polymers in food-contact environments were comprehensively assessed in this study. Important data regarding the stability and efficacy of food-grade thermochromic polymer blends in food packaging applications were obtained by subjecting them to six chemical solutions.

The results revealed that oxidative and acidic solutions affected the material’s absorption properties. The thermal cycling results showed that the most significant reductions were caused by bleach solution and hydroalcoholic solution, which resulted in minor activation temperature shifts in chemically exposed samples. The highest stability was demonstrated by HDPE100/TP0 in all conditions, as evidenced by tensile and impact strength testing. At the same time, HDPE98/TP2 and HDPE92/TP8 showed minor but measurable reductions in mechanical performance, particularly after prolonged exposure to acidic and oxidative environments.

While HDPE98/TP2 and HDPE92/TP8 showed greater vulnerability to chemically induced alterations, particularly under acidic and oxidative environments, HDPE100/TP0 showed the highest overall chemical resilience. Further stabilization methods are required to extend the shelf life of thermochromic masterbatch-containing polymers in food packaging to improve food safety monitoring and preserve material integrity. Several strategies could be considered to improve the chemical resistance of thermochromic polymers, including enhanced pigment encapsulation, incorporation of protective multilayer barriers, the use of compatibilizers and stabilizers targeting acid and oxidative degradation, and the selection of more chemically resilient pigment systems. The overall findings indicate that while HDPE98/TP2/HDPE98/TP8 remained chemically durable, the TP may have introduced some vulnerability under acidic, aqueous acidic, and alkaline solutions conditions.

This study provided a novel understanding of how different food-related chemical environments impact thermochromic functionality and mechanical durability. Through careful examination of color change, tensile and impact characteristics, mass and moisture stability, and microstructural integrity, it provided crucial information for maximizing thermochromic polymers for commercial food packaging. The insights gained contribute to bridging the gap between laboratory material development and real-world food safety and quality monitoring solutions.

The study demonstrates that recyclable HDPE, combined with thermochromic pigments, can be used as a sustainable packaging matrix for waste reduction and improved food safety. Although it has performance trade-offs, it is suitable for non-load-bearing components that may meet market demand for environmentally responsible packaging solutions.

The developed thermochromic blends also demonstrated compatibility with existing food packaging technologies. Using a food-grade HDPE matrix and standard injection molding processes without requiring equipment changes suggested that these materials could be incorporated into smart packaging applications alongside current production systems.

While this study demonstrated thermochromic polymer blends’ mechanical and optical durability in simulated food-contact environments, several limitations remained concerning their direct use in food packaging. Potential risks included pigment migration, encapsulation breakdown under acidic or oxidative exposure, and long-term loss of thermochromic functionality. These issues could compromise both product integrity and consumer safety. Therefore, future research should include migration testing following food safety regulations and a deeper evaluation of thermochromic systems’ encapsulation stability and regulatory compliance intended for direct-contact applications.

In parallel, future studies will also focus on integrating thermochromic systems into biodegradable polymer matrices and evaluating their degradation behavior under composting and environmental exposure conditions, supporting the development of sustainable smart packaging solutions.

Beyond mechanical durability and thermochromic responsiveness, future studies should also examine sensory compatibility and compositional interactions between packaging materials and food. This is particularly important in light of the observed surface deterioration, pigment fading, and minor mass increases under acidic and oxidative conditions. While the present work provides a strong foundation for the functional performance of thermochromic polymers, confirming their safety and suitability for intelligent food packaging will require further validation through standardized toxicological and regulatory assessments.

Additionally, the long-term effects of thermochromic polymer degradation—including potential pigment or additive migration, loss of temperature indication functionality, and surface deterioration—could impact food quality, safety, and shelf life. These factors highlight the need for extended stability testing, migration analysis under food-simulating conditions, and sensory evaluation to fully validate the safe use of thermochromic polymers in direct food-contact packaging applications.

## Figures and Tables

**Table 1:** Blend compositions prepared for this study.

Sample Name	HDPE (wt. %)	TP (wt. %)
HDPE100/TP0	100	0
HDPE98/TP2	98	2
HDPE92/TP8	92	8

**Table 2:** Solution type, test substance, concentration, measured pH, and exposure time for chemical resistance testing.

Solution Type	Test Substance	Concentration (%)	Measured pH	Exposure Time (h)
Acidic	Lemon juice	100%	2.3	0, 24, 48, 72
Acidic/Aqueous	Orange juice	100%	3.8	0, 24, 48, 72
Neutral Solution	Tap water	100%	7.0	0, 24, 48, 72
Solvent-Based	Hydroalcoholic solution	40%	7.2	0, 24, 48, 72
Oil-based Solution	Vegetable oil	100%	N/A	0, 24, 48, 72
Alkaline Solution	Bleach solution	5%	12.5	0, 24, 48, 72

**Table 3:** Total color difference (${\Delta E}_{ab}^{*}$) for thermochromic polymer specimens after exposure to food-contact solutions. Results are presented as mean ± standard deviation based on five replicate specimens per condition (*n* = 5).

Specimen ID	Solution Type	${\Delta E}_{ab}^{*}$ 24 h (Mean ± SD)	${\Delta E}_{ab}^{*}$ 48 h (Mean ± SD)	${\Delta E}_{ab}^{*}$ 72 h (Mean ± SD)
HDPE100/TP0	Lemon juice	0.42 (±0.03)	1.61 (±0.09)	1.73 (±0.04)
	Orange juice	0.48 (±0.02)	1.64 (±0.07)	1.89 (±0.09)
	Tap water	0.11 (±0.01)	0.19 (±0.01)	0.23 (±0.02)
	Hydroalcoholic solution	0.22 (±0.09)	0.31 (±0.07)	0.42 (±0.05)
	Vegetable oil	0.27 (±0.03)	0.61 (±0.09)	0.73 (±0.04)
	Bleach solution	0.40 (±0.11)	1.51 (±0.06)	1.66 (±0.03)
HDPE98/TP2	Lemon juice	0.92 (±0.11)	1.61 (±0.14)	1.94 (±0.19)
	Orange juice	1.05 (±0.13)	1.64 (±0.17)	2.09 (±0.21)
	Tap water	0.19 (±0.09)	0.27 (±0.11)	0.42 (±0.12)
	Hydroalcoholic solution	0.38 (±0.12)	0.50 (±0.18)	0.62 (±0.22)
	Vegetable oil	0.42 (±0.23)	0.61 (±0.19)	0.93 (±0.14)
	Bleach solution	0.83 (±0.21)	1.46 (±0.22)	1.73 (±0.19)
HDPE92/TP8	Lemon juice	1.21 (±0.11)	2.52 (±0.22)	3.54 (±0.32)
	Orange juice	1.37 (±0.12)	2.76 (±0.17)	3.75 (±0.29)
	Tap water	0.31 (±0.11)	0.45 (±0.11)	0.51 (±0.12)
	Hydroalcoholic solution	0.23 (±0.09)	0.42 (±0.12)	0.98 (±0.21)
	Vegetable oil	0.69 (±0.11)	0.87 (±0.19)	0.95 (±0.14)
	Bleach solution	1.11 (±0.13)	2.34 (±0.21)	3.30 (±0.19)

**Table 4:** The effect of exposure to five different test substances on the moisture content of HDPE100/TP0, HDPE98/TP2, and HDPE92/TP8 after 0, 24, 48, and 72 h. Color change values are presented as mean ± standard deviation based on five replicate specimens per condition (*n* = 5).

Specimen ID.	Test Substance	Moisture Content (%) (0 Exposure) (Mean ± SD)	Moisture Content (%) (After 24 h) (Mean ± SD)	Moisture Content (%) (After 48 h) (Mean ± SD)	Moisture Content (%) (After 72 h) (Mean ± SD)
HDPE100/TP0	Control	0.02 (±0.01)	0.02 (±0.01)	0.02 (±0.01)	0.02 (±0.01)
	Lemon juice	0.02 (±0.02)	0.05 (±0.02)	0.09 (±0.02)	0.12 (±0.02)
	Orange juice	0.02 (±0.02)	0.07 (±0.02)	0.12 (±0.02)	0.15 (±0.02)
	Tap water	0.02 (±0.01)	0.04 (±0.01)	0.06 (±0.01)	0.08 (±0.01)
	Hydroalcoholic solution	0.02 (±0.01)	0.02 (±0.01)	0.06 (±0.01)	0.08 (±0.01)
	Vegetable oil	0.02 (±0.01)	0.03 (±0.01)	0.07 (±0.01)	0.08 (±0.01)
	Bleach solution	0.02 (±0.02)	0.04 (±0.02)	0.08 (±0.02)	0.10 (±0.02)
HDPE98/TP2	Control	0.03 (±0.01)	0.03 (±0.01)	0.03 (±0.01)	0.03 (±0.01)
	Lemon juice	0.03 (±0.02)	0.07 (±0.01)	0.12 (±0.01)	0.15 (±0.01)
	Orange juice	0.03 (±0.02)	0.10 (±0.01)	0.16 (±0.01)	0.19 (±0.01)
	Tap water	0.03 (±0.01)	0.06 (±0.01)	0.08 (±0.01)	0.10 (±0.01)
	Hydroalcoholic solution	0.03 (±0.01)	0.03 (±0.01)	0.03 (±0.01)	0.03 (±0.01)
	Vegetable oil	0.03 (±0.01)	0.03 (±0.01)	0.03 (±0.01)	0.03 (±0.01)
	Bleach solution	0.03 (±0.02)	0.06 (±0.02)	0.10 (±0.03)	0.12 (±0.03)
HDPE92/TP8	Control	0.04 (±0.01)	0.04 (±0.01)	0.04 (±0.01)	0.04 (±0.01)
	Lemon juice	0.04 (±0.02)	0.09 (±0.02)	0.15 (±0.02)	0.18 (±0.02)
	Orange juice	0.04 (±0.02)	0.12 (±0.03)	0.19 (±0.03)	0.22 (±0.03)
	Tap water	0.04 (±0.01)	0.08 (±0.01)	0.10 (±0.01)	0.12 (±0.01)
	Hydroalcoholic solution	0.04 (±0.01)	0.07 (±0.01)	0.11 (±0.01)	0.12 (±0.04)
	Vegetable oil	0.04 (±0.01)	0.07 (±0.01)	0.10 (±0.01)	0.13(±0.03)
	Bleach solution	0.04 (±0.02)	0.08 (±0.02)	0.14 (±0.03)	0.16 (±0.04)

**Table 5:** The effect of different test substances on the mass of HDPE100/TP0, HDPE98/TP2, and HDPE92/TP8 after 0, 24, 48, and 72 h. Mass values are presented as mean ± standard deviation based on five replicate specimens per condition (*n* = 5).

Specimen ID.	Test Substance	Mass Measurement (g) (0 Exposure) (Mean ± SD)	Mass Measurement (g) (After 24 h) (Mean ± SD)	Mass Measurement (g) (After 48 h) (Mean ± SD)	Mass Measurement (g) (After 72 h) (Mean ± SD)
HDPE100/TP0	Control	3.724 (±0.00)	3.724 (±0.00)	3.724 (±0.00)	3.724 (±0.00)
	Lemon juice	3.724 (±0.02)	3.726 (±0.02)	3.736 (±0.03)	3.738 (±0.04)
	Orange juice	3.724 (±0.02)	3.727 (±0.04)	3.738 (±0.03)	3.741 (±0.05)
	Tap water	3.724 (±0.01)	3.725 (±0.01)	3.725 (±0.02)	3.725 (±0.01)
	Hydroalcoholic solution	3.724 (±0.01)	3.725 (±0.02)	3.726 (±0.01)	3.726 (±0.02)
	Vegetable oil	3.724 (±0.01)	3.725 (±0.02)	3.726 (±0.02)	3.726 (±0.02)
	Bleach solution	3.724 (±0.01)	3.725 (±0.02)	3.732 (±0.03)	3.735 (±0.04)
HDPE98/TP2	Control	3.724 (±0.00)	3.724 (±0.00)	3.724 (±0.00)	3.724 (±0.00)
	Lemon juice	3.724 (±0.02)	3.727 (±0.02)	3.730 (±0.03)	3.732 (±0.04)
	Bleach	3.724 (±0.02)	3.728 (±0.04)	3.737 (±0.03)	3.739 (±0.05)
	Tap water	3.724 (±0.01)	3.725 (±0.01)	3.727 (±0.02)	3.728 (±0.01)
	Hydroalcoholic solution	3.724 (±0.01)	3.724 (±0.02)	3.724 (±0.01)	3.725 (±0.02)
	Vegetable oil	3.724 (±0.01)	3.725 (±0.02)	3.725 (±0.02)	3.726 (±0.02)
	Bleach solution	3.724 (±0.02)	3.726 (±0.04)	3.729 (±0.03)	3.730 (±0.05)
HDPE92/TP8	Control	3.724 (±0.00)	3.724 (±0.00)	3.724 (±0.00)	3.724 (±0.00)
	Lemon juice	3.724 (±0.01)	3.728 (±0.02)	3.730 (±0.03)	3.733 (±0.04)
	Orange juice	3.724 (±0.02)	3.729 (±0.02)	3.732 (±0.03)	3.733 (±0.04)
	Tap water	3.724 (±0.01)	3.726 (±0.01)	3.727 (±0.02)	3.728 (±0.01)
	Hydroalcoholic solution	3.724 (±0.01)	3.725 (±0.02)	3.726 (±0.01)	3.727 (±0.02)
	Vegetable oil	3.724 (±0.01)	3.725 (±0.02)	3.726 (±0.02)	3.726 (±0.02)
	Bleach solution	3.724 (±0.02)	3.727 (±0.04)	3.729(±0.03)	3.731 (±0.05)

**Table 6:** The effect of different test substances on the activation temperature of HDPE100/TP0, HDPE98/TP2, and HDPE92/TP8 before and after 100 thermal cycles.

Specimen ID.	Test Substance	Initial Activation Temp. (°C)	Final Activation Temp. (°C)
HDPE98/TP2	Control	38.1	38.1
	Lemon juice	38.2	36.2
	Orange juice	38.2	35.5
	Tap water	38.3	37.9
	Hydroalcoholic solution	38.2	37.6
	Vegetable oil	38.0	37.5
	Bleach solution	38.2	36.4
HDPE92/TP8	Control	38.1	38.1
	Lemon juice	38.1	35.5
	Orange juice	38.4	34.2
	Tap water	38.3	37.6
	Hydroalcoholic solution	38.1	37.2
	Vegetable oil	38.2	36.9
	Bleach solution	38.3	35.9

**Table 7:** The effect of different test substances on the tensile strength of HDPE100/TP0 post 0, 24, 48 and 72 h. Values are presented as mean ± standard deviation based on five replicate specimens per condition (*n* = 5).

Specimen ID.	Test Substance	Time (h)	Tensile Strain (Displacement at Break) (%) (Mean ± SD)	Maximum Tensile Stress (MPa) (Mean ± SD)	Youngs Modulus (MPa) (Mean ± SD)
HDPE100/TP0	Lemon juice	0	90.27 (±7.89)	29.85 (±1.32)	785.83 (±12.30)
		24	85.26 (±11.23)	28.26 (±1.48)	741.55 (±14.41)
		48	80.04 (±12.31)	28.96 (±1.13)	716.80 (±11.32)
		72	103.29 (±13.76)	28.07 (±1.54)	710.20 (±13.85)
	Orange juice	0	100.84 (±7.11)	30.80 (±1.21)	743.49 (±12.23)
		24	110.05 (±12.97)	31.25 (±1.41)	708.60 (±12.11)
		48	85.27(±10.11)	32.24 (±1.56)	760.16 (±12.56)
		72	80.22 (±12.45)	33.30 (±1.43)	797.10 (±12.87)
	Tap water	0	80.24 (±7.76)	28.48 (±1.43)	741.57 (±13.21)
		24	100.41 (±8.56)	30.88 (±1.76)	715.07 (±11.99)
		48	101.05 (±9.98)	29.40 (±1.34)	782.69 (±12.54)
		72	100.36 (±8.09)	30.00 (±1.32)	786.18 (±11.13)
	Hydroalcoholic solution	0	86.73 (±7.09)	28.38 (±1.21)	727.93 (±11.83)
		24	100.84 (±8.11)	30.97 (±1.98)	808.31 (±11.54)
		48	95.61 (±10.12)	29.17 (±1.56)	704.57 (±12.12)
		72	90.73 (±11.59)	33.72 (±1.45)	797.33 (±12.89)
	Vegetable oil	0	100.38 (±8.12)	28.69 (±1.65)	749.32 (±13.32)
		24	80.22 (±11.67)	28.35 (±1.25)	761.54 (±14.01)
		48	100.37 (±12.21)	30.31 (±1.71)	748.11 (±12.03)
		72	95.49 (±9.11)	31.47 (±2.44)	750.82 (±13.72)
	Bleach solution	0	100.38 (±9.12)	28.69 (±1.87)	749.32 (±11.58)
		24	95.28 (±9.78)	29.61 (±1.94)	782.14 (±13.91)
		48	90.52(±8.99)	29.12 (±1.43)	742.75 (±14.32)
		72	85.56 (±11.45)	27.60 (±1.02)	694.36 (±13.98)

**Table 8:** The effect of different test substances on the tensile strength of P98/TP2 post 0, 24, 48 and 72 h. Values are presented as mean ± standard deviation based on five replicate specimens per condition (*n* = 5).

Specimen ID.	Test Substance	Time (h)	Tensile Strain (Displacement at Break) (%) (Mean ± SD)	Maximum Tensile Stress (MPa) (Mean ± SD)	Youngs Modulus (MPa) (Mean ± SD)
HDPE98/TP2	Lemon juice	0	95.75 (±7.89)	28.89 (±1.12)	647.29 (±13.11)
		24	100.55 (±7.31)	29.60 (±1.54)	755.85 (±14.23)
		48	96.47 (±7.64)	28.92 (±1.56)	766.31 (±14.51)
		72	90.45 (±7.19)	28.55 (±1.93)	756.33 (±13.25)
	Orange juice	0	100.32 (±8.49)	33.62 (±1.89)	816.49 (±14.23)
		24	95.24 (±9.34)	32.87 (±1.54)	784.96 (±12.65)
		48	96.01 (±10.24)	31.86 (±1.53)	765.22 (±12.13)
		72	102.00 (±11.32)	30.68 (±1.72)	688.22 (±13.45)
	Tap water	0	95.34 (±7.99)	28.01 (±1.89)	662.96 (±14.54)
		24	90.28 (±8.63)	29.16 (±1.31)	704.47 (±14.39)
		48	85.32 (±9.65)	31.58 (±1.65)	708.79 (±14.32)
		72	80.56 (±8.35)	31.61 (±1.42)	724.91 (±12.65)
	Hydroalcoholic solution	0	86.73 (±8.65)	28.38 (±1.89)	727.93 (±13.90)
		24	80.26 (±8.43)	31.42 (±1.68)	759.33 (±14.94)
		48	100.90 (±8.12)	28.03 (±1.75)	655.97 (±13.10)
		72	95.52 (±8.99)	30.24 (±1.24)	662.82 (±14.82)
	Vegetable oil	0	86.73 (±9.47)	28.38 (±1.48)	727.93 (±15.12)
		24	100.66 (±9.57)	28.66 (±1.02)	683.2 (±12.13)
		48	95.55 (±8.62)	28.66 (±1.49)	751.81 (±12.63)
		72	90.52 (±9.60)	31.67 (±1.56)	729.66 (±12.91)
	Bleach solution	0	80.48 (±10.49)	30.60 (±1.46)	707.51 (±13.12)
		24	114.13 (±11.94)	28.17 (±1.95)	721.67 (±13.34)
		48	100.62 (±10.34)	31.27 (±1.59)	751.92 (±13.89)
		72	90.52 (±9.87)	31.67 (±1.50)	729.67 (±13.24)

**Table 9:** The effect of different test substances on the tensile strength of HDPE92/TP8 post 0, 24, 48 and 72 h. Values are presented as mean ± standard deviation based on five replicate specimens per condition (*n* = 5).

Specimen ID.	Test Substance	Time (h)	Tensile Strain (Displacement at Break) (%) (Mean ± SD)	Maximum Tensile Stress (MPa) (Mean ± SD)	Youngs Modulus (MPa) (Mean ± SD)
PR92/TP8	Lemon juice	0	85.14 (±8.81)	27.77 (±1.21)	679.85 (±12.13)
		24	80.24 (±8.12)	30.02 (±1.18)	722.47 (±13.15)
		48	100.56 (±8.31)	28.63 (±1.17)	677.96 (±13.26)
		72	95.22 (±8.92)	32.83 (±1.16)	746.61 (±13.71)
	Orange juice	0	95.94 (±8.15)	27.62 (±1.15)	693.08 (±13.11)
		24	90.44 (±8.12)	28.96 (±1.72)	680.49 (±12.81)
		48	85.29 (±8.65)	30.96 (±1.64)	716.28 (±13.33)
		72	83.73 (±7.38)	28.64 (±1.27)	655.66 (±13.82)
	Tap water	0	100.44 (±7.19)	27.82 (±1.61)	711.64 (±13.10)
		24	96.15 (±8.89)	28.64 (±1.77)	715.85 (±13.27)
		48	100.35 (±7.12)	28.34 (±1.35)	769.71 (±13.93)
		72	95.64 (±7.38)	29.03 (±1.82)	768.21 (±13.41)
	Hydroalcoholic solution	0	101.00 (±7.89)	28.63 (±1.34)	729.54 (±13.11)
		24	103.65 (±7.23)	27.99 (±1.78)	603.05 (±13.31)
		48	92.97 (±7.15)	27.76 (±1.12)	679.39 (±13.14)
		72	85.87 (±7.79)	30.46 (±1.99)	769.73 (±13.79)
	Vegetable oil	0	90.27 (±7.19)	29.85 (±1.56)	785.83 (±13.88)
		24	85.25 (±7.68)	27.58 (±1.38)	715.51 (±14.76)
		48	80.45 (±7.57)	27.64 (±1.28)	744.91 (±14.45)
		72	102.98 (±7.29)	27.72 (±1.91)	741.50 (±14.13)
	Bleach solution	0	100.51 (±8.96)	30.82 (±1.56)	700.70 (±15.43)
		24	95.50 (±8.24)	28.75 (±1.82)	705.38 (±15.89)
		48	90.35 (±8.97)	27.90 (±1.93)	699.69 (±15.72)
		72	85.30 (±9.19)	29.64 (±2.26)	680.35 (±15.11)

**Table 10:** The effect of different test substances on the impact strength of HDPE100/TP0 with and without notches after chemical exposure post 0, 24, 48, and 72 h. Values are presented as mean ± standard deviation based on ten replicate specimens per condition (*n* = 10).

Specimen ID.	Test Substances	Time (h)	Impact Strength (kJ/m^2^) Unnotched (Mean ± SD)	Impact Strength (kJ/m^2^) Notched (Mean ± SD)
HDPE100/TP0	Lemon juice	0	99.63 (±1.82)	23.48 (±1.56)
		24	99.48 (±1.56)	23.35 (±1.34)
		48	99.38 (±1.55)	23.28 (±2.00)
		72	99.28 (±1.98)	23.15 (±2.56)
	Orange juice	0	99.43 (±1.46)	23.43 (±1.56)
		24	99.38 (±1.76)	23.28 (±1.65)
		48	99.30 (±2.09)	23.23 (±1.32)
		72	99.20 (±2.98)	23.10 (±2.86)
	Tap water	0	99.58 (±1.47)	23.44 (±1.45)
		24	99.43 (±1.95)	23.38 (±1.72)
		48	99.40 (±1.63)	23.37 (±1.88)
		72	99.38 (±1.79)	23.33 (±1.56)
	Hydroalcoholic solution	0	99.68 (±2.56)	23.36 (±1.06)
		24	99.63 (±2.99)	23.32 (±1.34)
		48	99.63 (±2.66)	23.28 (±1.32)
		72	99.60 (±2.56)	23.24 (±1.46)
	Vegetable oil	0	99.88 (±1.57)	23.33 (±1.21)
		24	99.80 (±1.88)	23.26 (±1.32)
		48	99.78 (±1.65)	23.21 (±1.11)
		72	99.70 (±1.70)	23.18 (±1.26)
	Bleach solution	0	99.75 (±1.57)	23.48 (±1.26)
		24	99.70 (±1.64)	23.37 (±1.45)
		48	99.60 (±1.87)	23.31 (±1.32)
		72	99.56 (±2.05)	23.21 (±2.13)

**Table 11:** The effect of different test substances on the impact strength of HDPE98/TP2 with and without notches after chemical exposure post 0, 24, 48, and 72 h. Values are presented as mean ± standard deviation based on ten replicate specimens per condition (*n* = 10).

Specimen ID.	Test Substance	Time (h)	Impact Strength (kJ/m^2^) Unnotched (Mean ± SD)	Impact Strength (kJ/m^2^) Notched (Mean ± SD)
HDPE98/TP2	Lemon juice	0	99.68 (±1.87)	23.48 (±2.56)
		24	99.40 (±1.99)	23.35 (±1.34)
		48	99.30 (±1.88)	23.28 (±2.00)
		72	99.18 (±2.10)	22.77 (±2.56)
	Orange juice	0	99.48 (±2.11)	23.44 (±2.42)
		24	99.18 (±2.54)	23.21 (±2.16)
		48	99.20 (±2.96)	22.79 (±2.57)
		72	99.13 (±2.56)	22.12 (±3.06)
	Tap water	0	99.60 (±1.80)	23.44 (±1.36)
		24	99.55 (±2.01)	23.38 (±1.75)
		48	99.48 (±2.29)	23.25 (±1.43)
		72	99.38 (±1.35)	23.13 (±1.20)
	Hydroalcoholic solution	0	99.48 (±2.56)	23.43 (±1.16)
		24	99.46 (±2.43)	23.16 (±1.22)
		48	99.40 (±2.42)	23.14 (±1.34)
		72	99.35 (±2.76)	23.02 (±1.97)
	Vegetable oil	0	99.65 (±2.67)	23.32 (±1.67)
		24	99.55 (±2.45)	23.22 (±1.98)
		48	99.52 (±2.90)	23.19 (±2.06)
		72	99.45 (±2.56)	23.14 (±2.33)
	Bleach solution	0	99.60 (±2.67)	23.31 (±2.46)
		24	99.50 (±2.84)	23.26 (±1.45)
		48	99.40 (±3.57)	23.12 (±2.64)
		72	99.25 (±2.89)	22.98 (±2.86)

**Table 12:** The effect of different test substances on the impact strength of HDPE92/TP8 with and without notches after chemical exposure post 0, 24, 48, and 72 h. Values are presented as mean ± standard deviation based on ten replicate specimens per condition (*n* = 10).

Specimen ID.	Test Substance	Time (h)	Impact Strength (kJ/m^2^) Unnotched (Mean ± SD)	Impact Strength (kJ/m^2^) Notched (Mean ± SD)
HDPE92/TP8	Lemon juice	0	99.38 (±2.03)	23.21 (±3.16)
		24	99.20 (±2.45)	22.71 (±3.29)
		48	99.11 (±2.54)	22.31 (±3.11)
		72	99.02 (±2.76)	22.14 (±3.86)
	Orange juice	0	99.08 (±2.54)	22.58 (±3.44)
		24	98.95 (±2.55)	22.11 (±3.66)
		48	98.55 (±2.78)	21.64 (±3.12)
		72	98.13 (±3.10)	21.02 (±3.96)
	Tap water	0	99.63 (±2.12)	23.34 (±2.35)
		24	99.58 (±2.93)	23.18 (±2.15)
		48	99.50 (±2.56)	23.02 (±2.05)
		72	99.50 (±2.74)	23.00 (±2.03)
	Hydroalcoholic solution	0	99.63 (±2.45)	23.12 (±1.06)
		24	99.58 (±2.63)	23.06 (±1.22)
		48	99.50 (±2.91)	23.04 (±1.34)
		72	99.48 (±2.44)	23.02 (±1.97)
	Vegetable oil	0	99.85 (±2.56)	23.45 (±1.97)
		24	99.80 (±2.76)	23.40 (±1.96)
		48	99.73 (±2.77)	23.29 (±2.31)
		72	99.68 (±2.94)	23.34 (±2.43)
	Bleach solution	0	99.85 (±3.13)	23.31 (±2.56)
		24	99.54 (±3.01)	23.06 (±1.54)
		48	99.23 (±3.54)	22.92 (±2.01)
		72	98.97 (±3.56)	22.64 (±2.86)

## Data Availability

The original contributions presented in this study are included in the article. Further inquiries can be directed to the corresponding author(s).
